# Yangjing Shugan decoction targets the Sirt1/Nrf2 antioxidant pathway and gut microbiota-metabolite axis to protect against premature ovarian failure

**DOI:** 10.3389/fphar.2025.1722692

**Published:** 2026-01-16

**Authors:** Ling Yang, Li Liu, Junbo Liu, Meng Ran Xu, Jing Ren, Bo Li, Lili Jiao, Yunyan Wei, Jing Wang, Yi Guo

**Affiliations:** 1 Affiliated Hospital Changchun University of Chinese Medicine, Changchun University of Chinese Medicine, Changchun, China; 2 Jilin Ginseng Academy, Changchun University of Chinese Medicine, Changchun, China; 3 School of Pharmaceutical Sciences, Changchun University of Chinese Medicine, Changchun, China; 4 College of Traditional Chinese Medicine, Changchun University of Chinese Medicine, Changchun, China; 5 The Third Affiliated Hospital of Changchun University of Chinese Medicine, Changchun University of Chinese Medicine, Changchun, China

**Keywords:** Yangjing Shugan decoction, SIRT1/NRF2, premature ovarian failure, antioxidant, gut microbiota

## Abstract

**Objective:**

This study aimed to assess the therapeutic potential of Yangjing Shugan decoction (YJSGD) in D-galactose (D-gal)-induced Premature Ovarian Failure (POF) mice and to elucidate its underlying mechanisms.

**Methods:**

The main metabolites in YJSGD were characterized. A D-gal-induced POF mouse model was established and intervened with YJSGD at doses of 25, 50, and 100 mg/kg. A comprehensive analysis encompassing ovarian function, oxidative stress, inflammation, the Sirt1/Nrf2 pathway, gut microbiota, short-chain fatty acids (SCFAs), and serum metabolomics was conducted.

**Results:**

The results demonstrated that YJSGD effectively restored estrous cyclicity, normalized serum estradiol (E2), follicle-stimulating hormone (FSH), and luteinizing hormone (LH) levels, and improved ovarian follicular development. YJSGD treatment also enhanced systemic antioxidant capacity and attenuated inflammation. Mechanistically, the therapeutic effects were associated with the upregulation of the Sirt1/Nrf2 signaling pathway in the ovary, as evidenced by increased protein expression of Sirt1, Nrf2, and HO-1, and suppressed Keap1. Furthermore, YJSGD ameliorated gut microbiota dysbiosis, promoted beneficial SCFAs production, and rectified serum metabolic disturbances involved in amino acid, lipid, and energy metabolism.

**Conclusion:**

The results indicate that YJSGD is a promising multi-target agent for POF treatment, and its synergistic effects on reproductive, oxidative, and gut microbiota homeostasis provide a solid basis for its clinical application.

## Graphical Abstract

**Figure:**
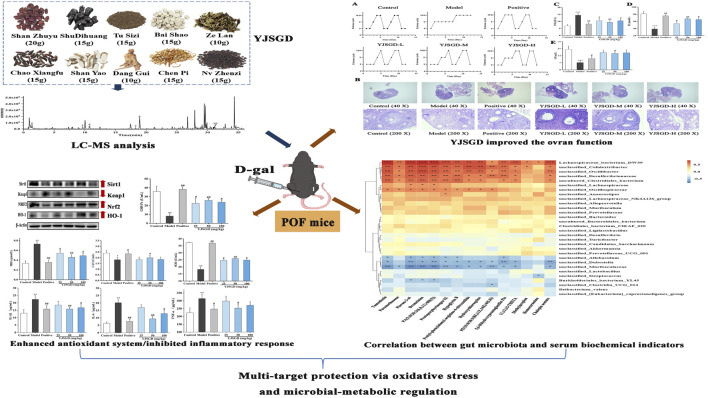

## 1 Introduction

Premature ovarian failure, characterized by diminished ovarian function before age 40, significantly compromises women’s reproductive health and quality of life (Poi et al., 2016). With an estimated incidence of 1%–5% among women of childbearing age and a trend toward younger onset (Jiao et al., 2018), POF represents a major challenge in reproductive medicine. Furthermore, it may cause infertility and associated health complications such as osteoporosis and cardiovascular disease (Huang et al., 2024b), posing substantial long-term health risks. Currently, the primary treatment for premature ovarian failure is hormone replacement therapy (HRT). While this therapy can alleviate symptoms of low estrogen levels, long-term use may increase the risk of breast cancer and blood clots, and it is difficult to fundamentally restore ovarian function (Huang et al., 2022). Consequently, developing safe and effective therapies capable of repairing ovarian function has attracted significant attention from scholars both domestically and internationally.

Oxidative stress is a key mechanism in cell damage, involves excessive reactive oxygen species (ROS) production that causes oxidative damage to cellular lipids, proteins, and DNA (Nouri et al., 2024). In ovarian granulosa cells, ROS accumulation impairs mitochondrial function, promotes apoptosis, disrupts estrogen secretion, and accelerates follicular atresia, ultimately contributing to POF. Concurrently, oxidative stress triggers pro-inflammatory factor overproduction, exacerbating ovarian injury. Traditional Chinese medicine (TCM) formulations, leveraging multi-component, multi-target advantages, show growing promise in treating complex diseases like POF (Huang et al., 2023). Critically, antioxidant activity constitutes a key therapeutic mechanism of TCM. These formulas often contain various antioxidants, such as flavonoids, saponins, polysaccharides, and polyphenols, which can exert antioxidant effects in multiple ways. Research has shown that granules primarily composed of American ginseng, notoginseng, and salvia miltiorrhiza delay D-gal-induced aging in mice by activating AMPK/SIRT1 to suppress oxidative stress and inflammation (Yin et al., 2025). TCM formulas are also gaining increasing attention in the treatment of premature ovarian failure. Si-Wu-Tang, which includes Angelica sinensis, Ligusticum chuanxiong, white peony root, and Rehmannia glutinosa, enhances ovarian function in cyclophosphamide-induced POF mice via Nrf2/HO-1 and STAT3/HIF-1α/VEGF pathways, enhancing antioxidant capacity and angiogenesis (Liu et al., 2023). Chaihu Shugan San, which includes *Rehmannia glutinosa*, *Cornus officinalis*, and other botanical drugs, effectively inhibits granulosa cell apoptosis in cyclophosphamide-induced POF rats by enhancing the activity of the PI3K/Akt/mTOR signaling pathway and modulating Bcl-2/Bax expression, thus protecting follicle integrity (Zeng et al., 2022). He’s Yangchao formula (HSYC) upregulates glutathione metabolism genes, including GPX8, GSTA1, and GSTA4, increases GSH, and reduces ROS through glutathione pathways, exerting anti-ovarian-aging effects (Yang et al., 2024). Huang et al. demonstrate that the kidney-tonifying and blood circulation-promoting (BHR) formula promoted bone marrow mesenchymal stem cells (BMSCs) proliferation via the SDF-1/CXCR4 and HGF/cMET signaling axes, thereby restoring ovarian reserve function and angiogenesis in cyclophosphamide-induced POI mice (Huang et al., 2024a).

The Yangjing Shugan Decoction used in this study is a Traditional Chinese Medicine formula comprising 10 traditional Chinese medicine, including *C. officinalis* Sieb. et Zucc., *R. glutinosa* (Gaertn.) DC., *Angelica sinensis* (Oliv.) Diels, *Paeonia lactiflora* Pall., *Dioscorea opposita* Thunb., *Citrus reticulata* Blanco, *Cuscuta chinensis Lam., Ligustrum lucidum* Ait., *Lycopus lucidus* Turcz. var. hirtus Regel and *Cyperus rotundus* L. With a long-standing history of clinical application, YJSGD demonstrates potential therapeutic efficacy against POF. However, its precise mechanisms of action remain unclear. To address this, the major metabolites of YJSGD were characterized by Ultra Performance Liquid Chromatography-Quadrupole-Time of Flight Mass Spectrometry (UPLC-Q-TOF/MS), evaluated its regulatory effects on ovarian function, antioxidant enzyme activity, and inflammatory factors in a D-gal-induced POF mouse model, and investigated its modulation of gut microbiota, microbial metabolites, and serum metabolomics via 16S rRNA sequencing and metabolomic analysis. This study provides scientific evidence supporting YJSGD as a potential therapeutic agent for POF by elucidating its pharmacodynamic material basis and mechanistic pathways.

## 2 Materials and methods

### 2.1 Experimental materials

SOD, CAT, GSH-Px and MDA assay kits were obtained from Nanjing Jiancheng Bioengineering Institute (Nanjing, China). The 4% paraformaldehyde fixative solution, NP40 lysis buffer, SDS-PAGE protein loading buffer (5X) were supplied by Beyotime Biotechnology (Shanghai, China). H&E staining kit was acquired from Solarbio Science and Technology Co., Ltd. (Beijing, China). ELISA kits for TNF-α, IL-1β, IL-6, LH, E2, and FSH were procured from Huangshi Yanke Biotechnology Co., Ltd. (Hubei, China). Chromatographic grade acetonitrile, methanol, and formic acid aqueous solution were supplied by ANPEL Laboratory Technologies Inc. (Shanghai, China). The BCA protein assay kit, rapid blocking buffer, and ECL substrate were obtained from New Cell and Molecular Biotech Co., Ltd. (Suzhou, China). Primary antibodies against β-actin, Nrf2, Keap1, HO-1, and Sirt1 were purchased from Boster Biological Technology Co., Ltd. (Wuhan, China). YJSGD comprises ten botanical drugs: *C. officinalis* Sieb. et Zucc. [Cornaceae; Corni Fructus], *R. glutinosa* Libosch. [Scrophulariaceae; Rehmanniae Radix Praeparata], *An*g*elica sinensis* (Oliv.)Diels [Umbelliferae; Angelicae Sinensis Radix], *P. lactiflora* Pall. [Ranunculaceae; Paeoniae Radix Alba], *D. opposita* Thunb. [Dioscoreaceae, Dioscoreae Rhizoma], *C. reticulata* Blanco [Rutaceae; Citri Reticulatae Pericarpium], *C. chinensis* Lam. [Convolvulaceae; Cuscutae Semen], *L. lucidum* Ait. [Oleaceae, Ligustri Lucidi Fructus], *L. lucidus* Turcz. var. *hirtus* Regel [Lamiaceae; Lycopi Herba], and *C. rotundus* L. [Cyperaceae; Cyperi Rhizome]. All materials, supplied by Jilin Guoa’n Pharmaceutical Co., Ltd. (Jilin, China), complied with the specifications of the Chinese Pharmacopoeia (2025). Further details are listed in Table 1.

**TABLE 1:** The composition of YJSGD.

Chinese name^a^	Family	Latin name^a^ ^,^ ^b^	Processing	Plant part	Botanical drugs (g)
Shanzhuyu	Cornaceae	*Cornus officinalis* Sieb. et Zucc	Dried	Ripe pulp	20
Shudihuang	Scrophulariaceae	*Rehmannia glutinosa* Libosch	Liquor processed	Root tuber	15
Danggui	Umbelliferae	*Angelica sinensis* (Oliv.) Diels	Dried	Root	10
Baishao	Ranunculaceae	*Paeonia lactiflora* Pall	Dried	Root	15
Shanyao	Dioscoreaceae	*Dioscorea opposita* Thunb	Dried	Rhizome	15
Chenpi	Rutaceae	*Citrus reticulata* Blanco	Dried	Pericarp	15
Tusizi	Convolvulaceae	*Cuscuta chinensis Lam*	Dried	Ripe Seed	15
Nvzhenzi	Oleaceae	*Ligustrum lucidum* Ait	Dried	Ripe fruit	15
Zelan	Lamiaceae	*Lycopus lucidus Turcz. var. hirtus Regel*	Dried	Aerial Parts	10
Chaoxiangfu	Cyperaceae	*Cyperus rotundus* L	Dried	Rhizome	15

^a^ According to the Pharmacopeia of China (2025).

^b^ The Latin names of the botanical drugs are found on the http://mpns.kew.org/mpns-portal.

### 2.2 Experimental method

#### 2.2.1 Preparation of YJSGD

**FIGURE 1:**
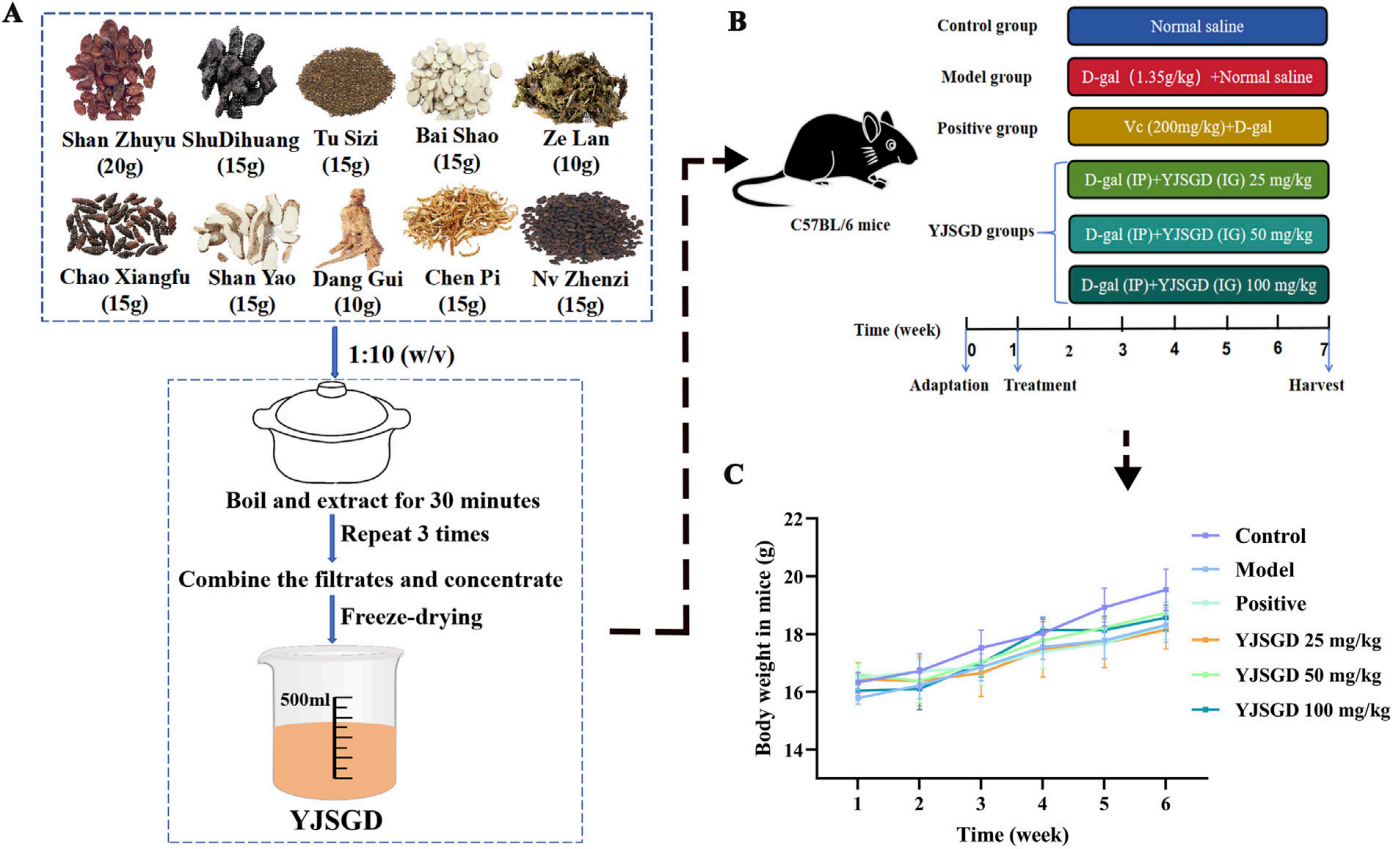
Extraction process of YJSGD **(A)**, its intervention on POF mice **(B)**, and effects on body weight **(C)**.

YJSGD was prepared using a total of 145 g of crude drugs (Figure 1A). The formulation consisted of ten botanical materials: Corni Fructus, Rehmanniae Radix Praeparata, Angelicae Sinensis Radix, Paeoniae Radix Alba, Dioscoreae Rhizoma, Citri Reticulatae Pericarpium, Cuscutae Semen, Ligustri Lucidi Fructus, Lycopi Herba, and Cyperi Rhizoma, in a ratio of 4:3:2:3:3:3:3:3:2:3. Briefly, the botanical drugs were decocted with distilled water (1:10, w/v) and extracted three times (30 min per extraction). The resulting filtrates were combined, concentrated by rotary evaporation, and lyophilized to obtain the dried extract with a yield of 30.72%. The extract was then reconstituted in distilled water to final concentrations of 25 mg/kg, 50 mg/kg, and 100 mg/kg (w/v) for oral gavage administration in mice.

#### 2.2.2 Identification of major metabolites of YJSGD by UPLC-Q/TOF-MS analysis

The major metabolites in YJSGD were analyzed using ultra-high performance liquid chromatography coupled with UPLC-Q-TOF-MS, with an Agilent ZORBAX SB-Aq column (2.1 × 100 mm, 1.8 µm). The flow rate was set at 0.3 mL/min, and the sample injection volume was 2 μL. The mobile phase consisted of acetonitrile (A) and 0.1% formic acid aqueous solution (B). The multi-step linear elution gradient program was as follows: 0–5 min, 0%–10% A; 5–13 min, 10%–15% A; 13–24 min, 15%–28% A; 24–30 min, 28%–70% A; 30–33 min, 70%–95% A; 33–36 min, 95% A; 36–36.1 min, 95%–0% A; 36.1–39 min, 0% A.

MS and MS/MS data were acquired using an AB Sciex TripleTOF® 4600 SCIEX coupled with Analyst TF 1.7.1 and PeakView 1.2 software in ESI positive/negative switching mode. Instrument parameters were set as follows: TOF mass range 50–1700 Da, ion source gases 1 and 2 at 50 psi, curtain gas 35 psi, ion spray voltage ±5000 V, source temperature 500 °C, and declustering potential 100 V. For MS/MS acquisition, the mass range was 50–1,250 Da with declustering potential 100 V, collision energy 40 eV (fixed), collision energy spread 20 eV, ion release delay 30 ms, and ion release width 15 ms.

#### 2.2.3 Protect against premature ovarian failure of YJSGD

##### 2.2.3.1 Animals and treatment

Following a 1-week acclimatization period, 7-week-old female C57BL/6 mice were randomly divided into six groups: Control group, Model group, Positive group, YJSGD-L group (25 mg/kg), YJSGD-M group (50 mg/kg), and YJSGD-H group (100 mg/kg). All groups except control group received daily intraperitoneal injections of D-gal (1.35 g/kg), while model group received 0.2 mL saline (oral gavage), positive group received 0.2 mL vitamin C (200 mg/kg, oral gavage), and YJSGD groups received corresponding YJSGD doses; concurrently, control group received equivalent saline via both intraperitoneal injection and oral gavage (Figure 1B). All procedures strictly adhered to China’s State Council Regulations on Laboratory Animal Administration with approval from the Animal Ethics Committee of Changchun University of Chinese Medicine (Approval No.: 2022612). Body weights were monitored throughout the experimental period. At sacrifice, the liver, kidneys, spleen, and ovaries were excised, weighed, and their organ indices calculated as: organ index (%) = [organ wet weight (mg)/body weight (g)] × 100.

##### 2.2.3.2 Biochemical index detection

Kits from the Nanjing Institute of Biological Engineering were used to measure the activity of SOD, GSH-Px, CAT, and MDA in serum according to the manufacturer’s instructions. Serum concentrations of FSH, LH, E2, IL-1β, IL-6, and TNF-α were measured using enzyme-linked immunosorbent assay (ELISA) kits, following the manufacturer’s protocols.

##### 2.2.3.3 Estrous cycle monitoring

For 12 consecutive days prior to study termination, vaginal smears were collected daily between 08:00–09:00 using saline lavage. The lavage fluid was mounted on glass slides, air-dried, and stained with Wright’s solution. Estrous cycle stages (proestrus, estrus, metestrus, diestrus) were determined by microscopic examination of cell morphology (Cora et al., 2015).

##### 2.2.3.4 Examination of ovarian histopathology

Ovarian tissues were fixed in 4% paraformaldehyde for 24 h after collection. Following routine dehydration and paraffin embedding, serial sections of 4–5 μm thickness were prepared. H&E staining was performed according to the manufacturer’s protocol for the staining kit. Subsequently, stained sections were observed under a microscope and images were captured.

##### 2.2.3.5 Western blot analysis

Western blotting was performed to quantify ovarian expression of Nrf2, Keap1, HO-1, and Sirt1 in an ovarian failure mouse model, with β-actin as the internal reference. Proteins were extracted using NP-40 lysis buffer, and their concentrations were determined by BCA method. Protein lysates were then subjected to SDS-PAGE electrophoresis, transferred to PVDF membranes, and blocked with 5% non-fat milk. Membranes were probed with primary antibodies at 4 °C overnight, washed with TBST, then incubated with HRP-conjugated secondary antibodies. Protein bands were visualized using ECL reagent chemiluminescence.

##### 2.2.3.6 Analysis of intestinal microbiota

Under sterile conditions, fecal samples were collected for subsequent analysis. To detect differences in the gut microbiota between samples, we conducted α diversity and β diversity analyses. For α diversity, Vegan was used to assess the richness and diversity of each sample. For β diversity, principal coordinate analysis (PCoA) and analysis of similarities (ANOSIM) were employed to evaluate the Bray-Curtis dissimilarity index.

##### 2.2.3.7 Determination of SCFAs in mouse feces

SCFAs levels were quantified by GC-MS using a Trace 1310-TSQ 8000 system equipped with a TR-FFAP capillary column (30 m × 0.25 mm × 0.25 μm). Analysis was performed with a flame ionization detector (FID) under the following parameters: injection port temperature 230 °C with 1.0 μL injection volume, ion source temperature 280 °C, and helium carrier gas at 1.0 mL/min flow rate with 10:1 split ratio. The oven temperature program initiated at 100 °C (hold 1 min), increased to 160 °C at 5 °C/min (hold 5 min), then ramped to 200 °C at 25 °C/min (hold 2 min). SCFAs standards (acetic, propionic, butyric acids, isobutyric acid, valeric acid, isovaleric acid, and hexanoic acid; Sigma-Aldrich) were used for quantification.

##### 2.2.3.8 Untargeted metabolomics analysis of mouse serum

100 µL of serum was deproteinized with 300 µL of cold methanol/acetonitrile (1:1, v/v), vortexed, and centrifuged. The supernatant was dried under nitrogen, reconstituted in ultrapure water, and filtered. A pooled quality control (QC) sample was generated from all aliquots. Chromatographic separation was performed on a Thermo Accucore C18 column (50 × 2.1 mm, 1.9 µm) at 35 °C using a gradient of 0.1% formic acid in water (A) and acetonitrile (B) at 0.3 mL/min. The injection volume was 2.0 µL. Mass spectrometry analysis was conducted on a Ultra-High Performance Liquid Chromatography Quadrupole-Orbitrap Mass Spectrometry (UHPLC-Q-Exactive Orbitrap/MS) system with an ESI source operating in both positive and negative full MS/dd-MS^2^ mode. System stability was monitored by analyzing blank and QC samples throughout the run. Data were acquired and processed using Xcalibur software.

## 3 Results

### 3.1 Characterization of YJSGD metabolites by UPLC-Q-TOF-MS

UPLC-Q-TOF-MS analysis was used to identify the major chemical metabolites in YJSGD. By using both positive and negative ion modes for dual detection, we ensured a comprehensive analysis of the YJSGD extract (Supplementary Figures S1, S2; Table 2). By integrating high-resolution natural product mass spectra databases with literature evidence, and correlating full-scan MS data, primary mass spectrum ion data, secondary collision energy, and fragment patterns, we structural characterized 10 meatbolites (Table 2): Rehmannioside D (81720–08-3), Morroniside (25406–64-8), Loganin (18524–94-2), Paeoniflorin (23180–57-6), Ferulic acid (1,135–24-6), Hyperoside (482–36-0), Specnuezhenide (449733–84-0), Hesperidin (520–26-3), Luteolin (491–70-3), and Ursolic acid (CAS: 77–52-1).

**TABLE 2:** Identification of the major metabolites in YJSGD by UPLC-Q-TOF-MS.

No.	Time (min)	Extraction mass	Formular	Metabolites	Origniation
1	4.55	731.2235	C_27_H_42_O_20_	Rehmannioside D	*Rehmannia glutinosa* Libosch
2	7.70	451.1456	C_17_H_26_O_11_	Morroniside	*Cornus officinalis* Sieb. et Zucc
3	9.66	435.1507	C_17_H_26_O_10_	Loganin	*Cornus officinalis* Sieb. et Zucc
4	10.75	525.1629	C_23_H_28_O_11_	Paeoniflorin	*Paeonia lactiflora* Pall
5	14.11	193.0499	C_10_H_10_O_4_	Ferulic acid	*Angelica sinensis* (Oliv.) Diels
6	17.12	463.0893	C_21_H_20_O_12_	Hyperoside	*Cuscuta chinensis* Lam
7	18.28	685.2356	C_31_H_42_O_17_	Specnuezhenide	*Ligustrum lucidum* Ait
8	18.95	609.1829	C_28_H_34_O_15_	Hesperidin	*Citrus reticulata* Blanco
9	25.11	285.0403	C_15_H_10_O_6_	Luteolin	*Cyperus rotundus* L
10	32.47	439.3556	C_30_H_48_O_3_	Ursolic acid	*Lycopus lucidus* Turcz. var. hirtus Regel

### 3.2 YJSGD increased the body weight and organ index of POF mice

We constructed a POF mouse model using continuous D-gal injections (Figure 1B) and monitored the changes in the mice’s body weight throughout the experiment. As shown in Figure 1C, during the entire experiment, the body weight of the control group mice increased steadily, whereas the model group mice experienced a slower weight gain compared to the control group. Following YJSGD treatment, the body weight gain in model mice approximated a trend comparable to that of the control group. Table 3 shows that, compared to the control group, the organ indices of the model group mice were significantly reduced (*p* < 0.05). Following YJSGD intervention, the organ indices of the mice increased, with the 50 mg/kg (YJSGD-M) dose showing the most significant effect.

**TABLE 3:** The effect of YJSGD on organ indices (Mean ± SD, n = 6).

Groups	Ovarian index (mg/g)	Spleen index (mg/g)	Liver index (mg/g)	Kidney index (mg/g)
Control	0.43 ± 0.05	3.53 ± 0.40	44.22 ± 2.47	12.89 ± 0.44
Model	0.31 ± 0.04^*^	2.82 ± 0.27^*^	37.53 ± 2.4^*^	9.88 ± 0.70^*^
Positive	0.37 ± 0.06^#^	3.48 ± 0.30^#^	43.72 ± 3.18^#^	11.28 ± 0.55^#^
YJSGD-25	0.33 ± 0.04	3.29 ± 0.40	41.62 ± 2.69^#^	10.64 ± 0.87
YJSGD-50	0.39 ± 0.03^#^	3.35 ± 0.55^#^	42.54 ± 1.23^#^	11.10 ± 1.01^#^
YJSGD-100	0.36 ± 0.06^#^	3.27 ± 0.23	40.21 ± 2.50	10.90 ± 0.81^#^

* *P* < 0.05, Model group vs. Control group.

# *P* < 0.05, YJSGD, group vs. Model group.

### 3.3 YJSGD improved estrous cyclicity and ovarian morphology in POF mice

**FIGURE 2:**
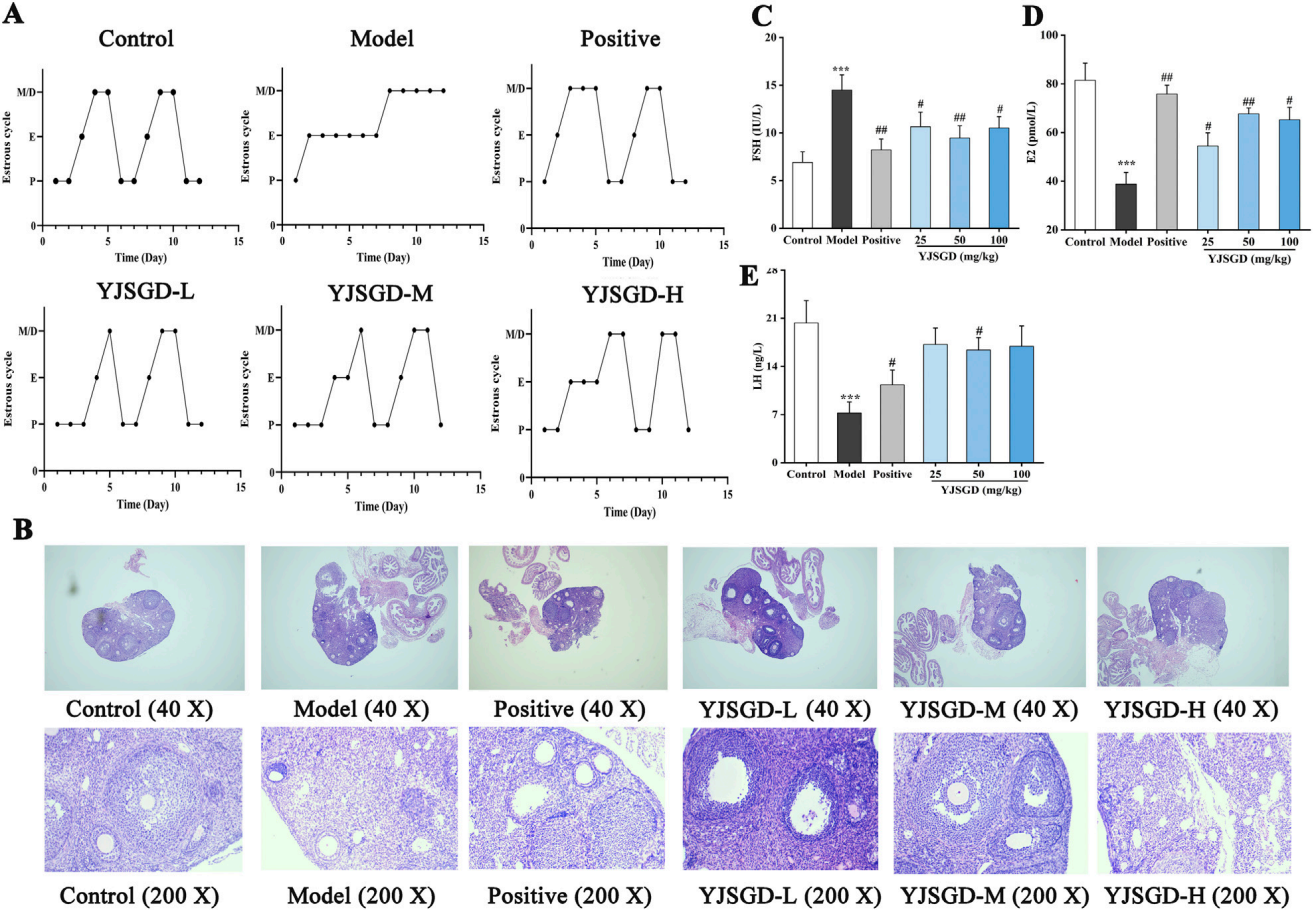
YJSGD improved the ovran function of POF mice. **(A)** Vaginal smear analysis of estrous cycle stages. **(B)** Ovarian histomorphology (H&E staining, Top: ×40 magnification (Scale bar: 200 μm); Bottom: ×200 magnification (Scale bar: 200 μm). **(C–E)** Serum hormone levels in POF mice. Data are displayed as means ± SD, n = 6. ^*^ *p* < 0.05, ^**^ *p* < 0.01 vs. the control group. ^#^ *p* < 0.05, ^##^ *p* < 0.01 vs. the model group.

As shown in Figure 2A, continuous D-gal injections caused significant disruption to the estrous cycle of mice. However, YJSGD intervention helped restore the regularity of the estrous cycle in POF mice. To further investigate the effect of YJSGD on ovarian function in POF mice, we performed H&E staining of ovarian tissue to observe its impact on follicular development. As shown in Figure 2B, the model group exhibited a reduction in follicles, disordered arrangement of granulosa cells, and an increase in atretic follicles. YJSGD significantly improved these conditions in POF mice, with the intermediate dose group showing the best improvement in ovarian tissue damage.

### 3.4 YJSGD improved hormone levels in POF mice

As shown in Figures 2C–E, compared to the control group, the model group showed a significant increase in FSH levels (*p* < 0.01) and a significant decrease in E2 and LH concentration (*p* < 0.01). In contrast, after administration of YJSGD, the serum FSH levels in POF mice decreased significantly (*p* < 0.01, *p* < 0.05), and the E2 and LH level increased significantly (*p* < 0.01, *p* < 0.05). Overall, YJSGD effectively improved the estrous cycle disorder in POF mice, reversed abnormal hormone levels, and the YJSGD-M group showed the best result, indicating that YJSGD can effectively improve the ovarian function of POF mice.

### 3.5 YJSGD enhances the antioxidant and anti-inflammatory capabilities of POF mice

**FIGURE 3:**
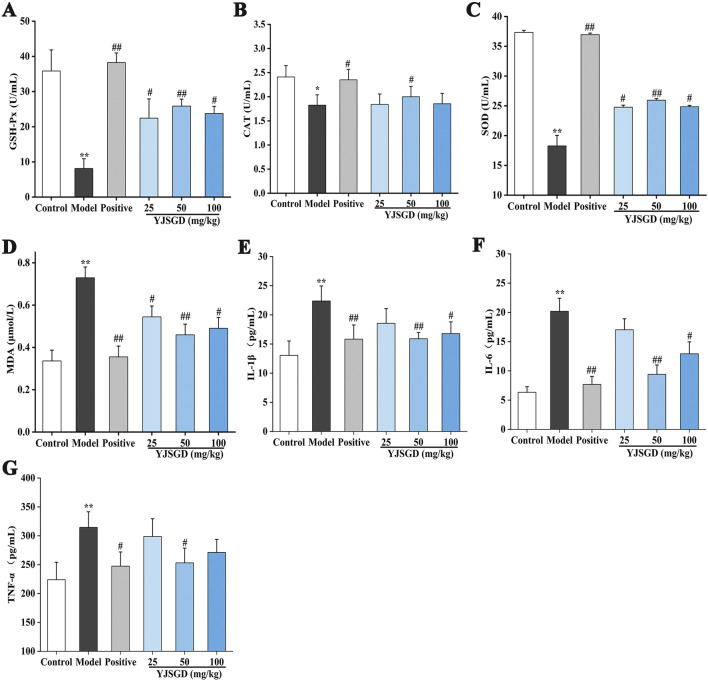
YJSGD enhanced the antioxidant system **(A–D)** and inhibited inflammatory response **(E–G)** of POF mice. Data are displayed as means ± SD, n = 6. ^*^ *p* < 0.05, ^**^ *p* < 0.01 vs. the control group. ^#^ *p* < 0.05, ^##^ *p* < 0.01 vs. the model group.

As illustrated in Figures 3A–D, serum MDA levels were significantly elevated in the model group of mice compared to the control group (*p* < 0.001), whereas CAT, SOD, and GSH-Px activities were significantly reduced (*p* < 0.05, *p* < 0.001). YJSGD intervention significantly reduced MDA levels and enhanced CAT, SOD and GSH-Px activities in POF mice (*p* < 0.05, *p* < 0.01). Notably, the YJSGD-M group exhibited significant increased antioxidant activity (*p* < 0.05, *p* < 0.01). These results demonstrate that YJSGD, particularly at the medium dose, effectively ameliorates oxidative damage in D-gal induced POF mice through upregulation of antioxidant enzymes and suppression of lipid peroxidation.

As demonstrated in Figures 3E–G, serum levels of IL-1β, IL-6 and TNF-α were significantly increased in the model group compared with the control group (*p* < 0.01). YJSGD intervention at medium (YJSGD-M) and high dose (YJSGD-H) significantly reduced IL-1β and IL-6 levels (*p* < 0.05, *p* < 0.01). Notably, TNF-α levels were markedly attenuated in the YJSGD-M group (*p* < 0.05).

### 3.6 YJSGD activated sirt1/Keap1/Nrf2/HO-1 pathway

**FIGURE 4:**
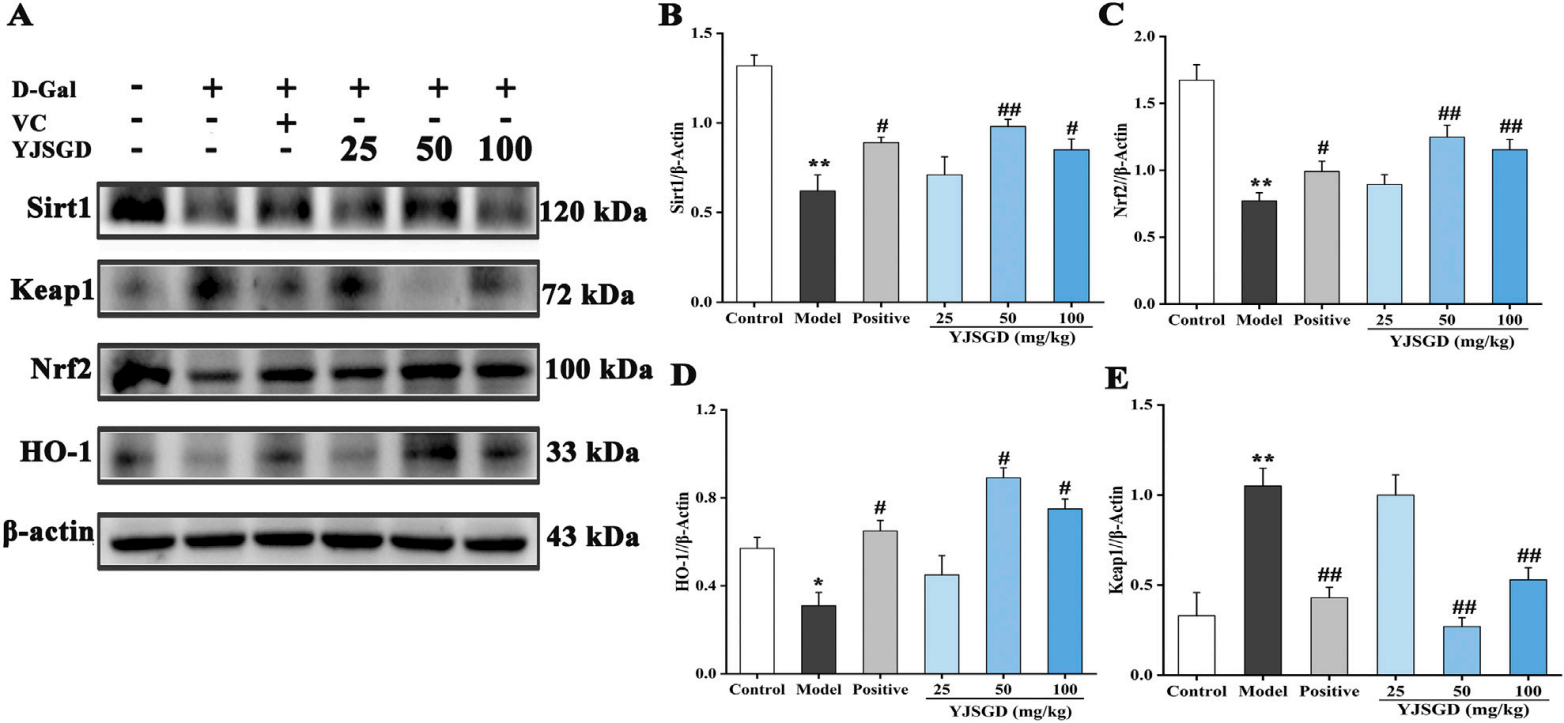
YJSGD enhanced the expression of related proteins. The expression **(A)** and quantitative analysis **(B–E)** of the related proteins in ovary. Data are displayed as means ± SD, n = 6. ^*^ *p* < 0.05, ^**^ *p* < 0.01 vs. the control group. ^#^ *p* < 0.05, ^##^ *p* < 0.01 vs. the model group.

Western blot analysis (Figure 4) showed significantly reduced expression of Sirt1, Nrf2, and HO-1 proteins in ovarian tissues of POF mice compared to control group (*p* < 0.05, *p* < 0.01), alongside increased Keap1 expression. YJSGD intervention significantly upregulated the expression of Sirt1, Nrf2, and HO-1 and downregulated Keap1 expression (*p* < 0.05, *p* < 0.01). Our findings are consistent with the idea that YJSGD enhances antioxidant defenses and ameliorates ovarian dysfunction, potentially by implicating the Sirt1-mediated Keap1-Nrf2-HO-1 signaling pathway, though future studies with Sirt1 inhibitors are needed to establish causality.

### 3.7 YJSGD improved the intestinal microbiota and SCFAs levels in POF mice

#### 3.7.1 Diversity analysis

**FIGURE 5:**
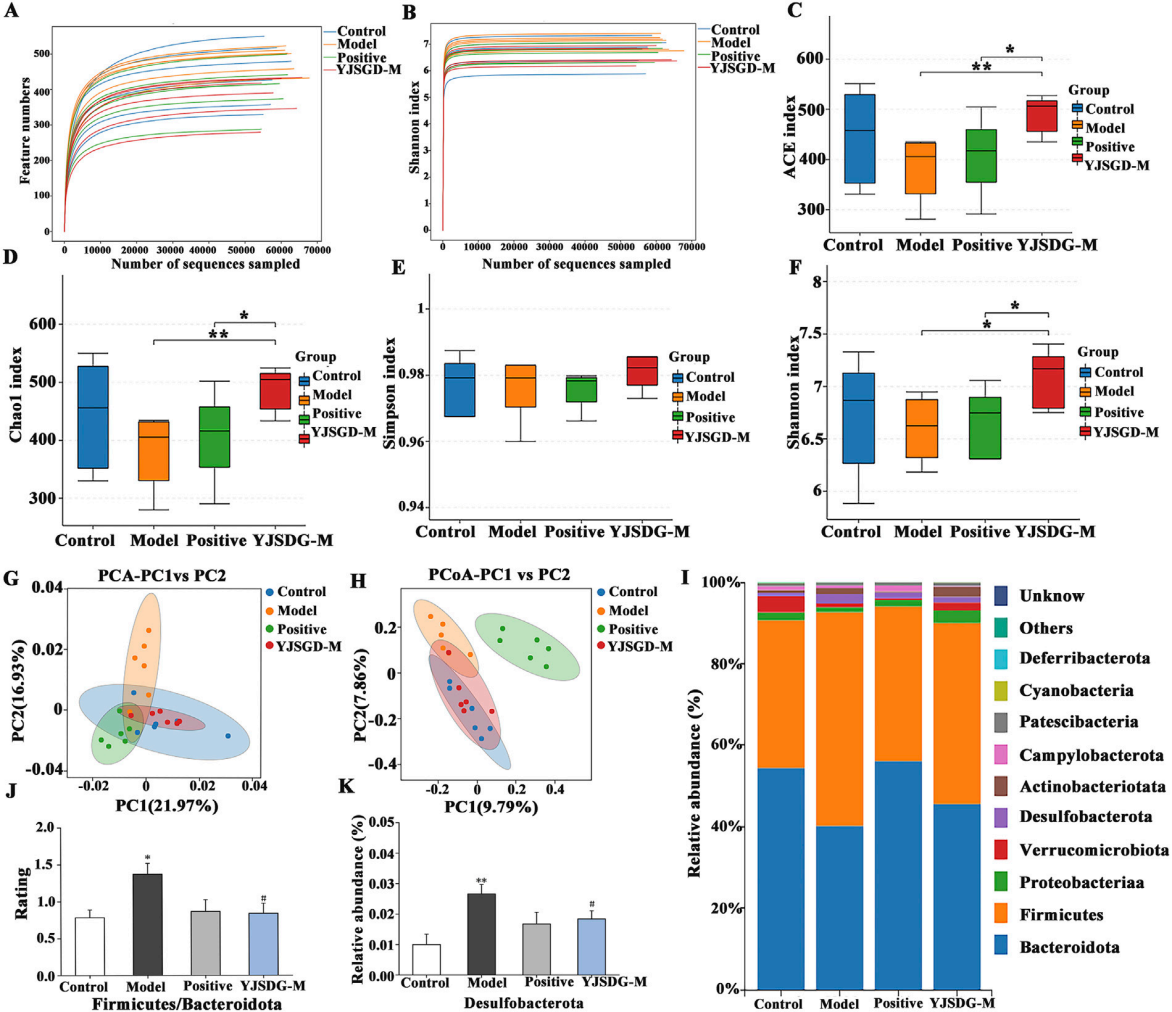
YJSGD alters fecal microbiota composition in POF mice. Alpha diversity analysis (**(A)**: Dilution curve; **(B)** Shannon curve; **(C)** ACE; **(D)** Chao1; **(E)** Simpson index; **(F)** Shannon index); Beta diversity analysis **(G)**: PCA; **(H)** PCoA). Phylum-level bar plot **(I)** and signature bacteria analysis across treatment groups **(J–K)**. Data are displayed as means ± SD, n = 6. ^*^ *p* < 0.05, ^**^ *p* < 0.01 vs. the control group. ^#^ *p* < 0.05, ^##^ *p* < 0.01 vs. the model group.

16S rRNA gene sequencing was used to assess gut microbiota diversity. As shown in Figures 5A,B, the rarefaction curve approaches a plateau with increasing sequencing effort, indicating that the sequence quantity per sample meets requirements for data analysis. Similarly, the stabilization of the Shannon diversity curve confirms adequate sequencing depth, capturing the vast majority of microbial diversity information within samples. Alpha diversity analysis (Figures 5C–F) indicates that the ACE, Chao1, Simpson and Shannon indices of the YJSGD-M group are significantly higher than those of the model group (*p* < 0.01, *p* < 0.05), suggesting a higher species richness in the YJSGD-M group. Additionally, beta diversity analysis based on PCA and PCoA reveals that the microbial community structure of the YJSGD-M group was significantly different from that of the model group (Figures 5G,H). Specifically, PC1 and PC2 of PCA explain 21.97% and 16.93% of the variation, respectively, while PCoA explains 9.79% and 7.86% of the variation, respectively. These results confirmed that YJSGD significantly altered the composition of the gut microbiota in POF mice.

#### 3.7.2 YJSGD improves the intestinal flora composition of POF mice at the phylum and genus levels in POF mice

Dysbiosis of the intestinal flora can exacerbate ovarian dysfunction. As shown in Figure 5I; Supplementary Figure S2, at the phylum level, the control group is dominated by Firmicutes and Bacteroidetes. In contrast, the F/B ratio in the model group of mice is significantly higher, with an increased abundance of Desulfobacterota and a decrease in Proteobacteria and Verrucomicrobia. After YJSGD intervention, the F/B ratio (Figure 5J) in POF mice is significantly reduced, the abundance of Desulfobacterota (Figure 5K) decreases, and the abundance of Actinobacteria increases. These results suggest that YJSGD can effectively reverse the family-level imbalance of the intestinal flora induced by POF.

**FIGURE 6:**
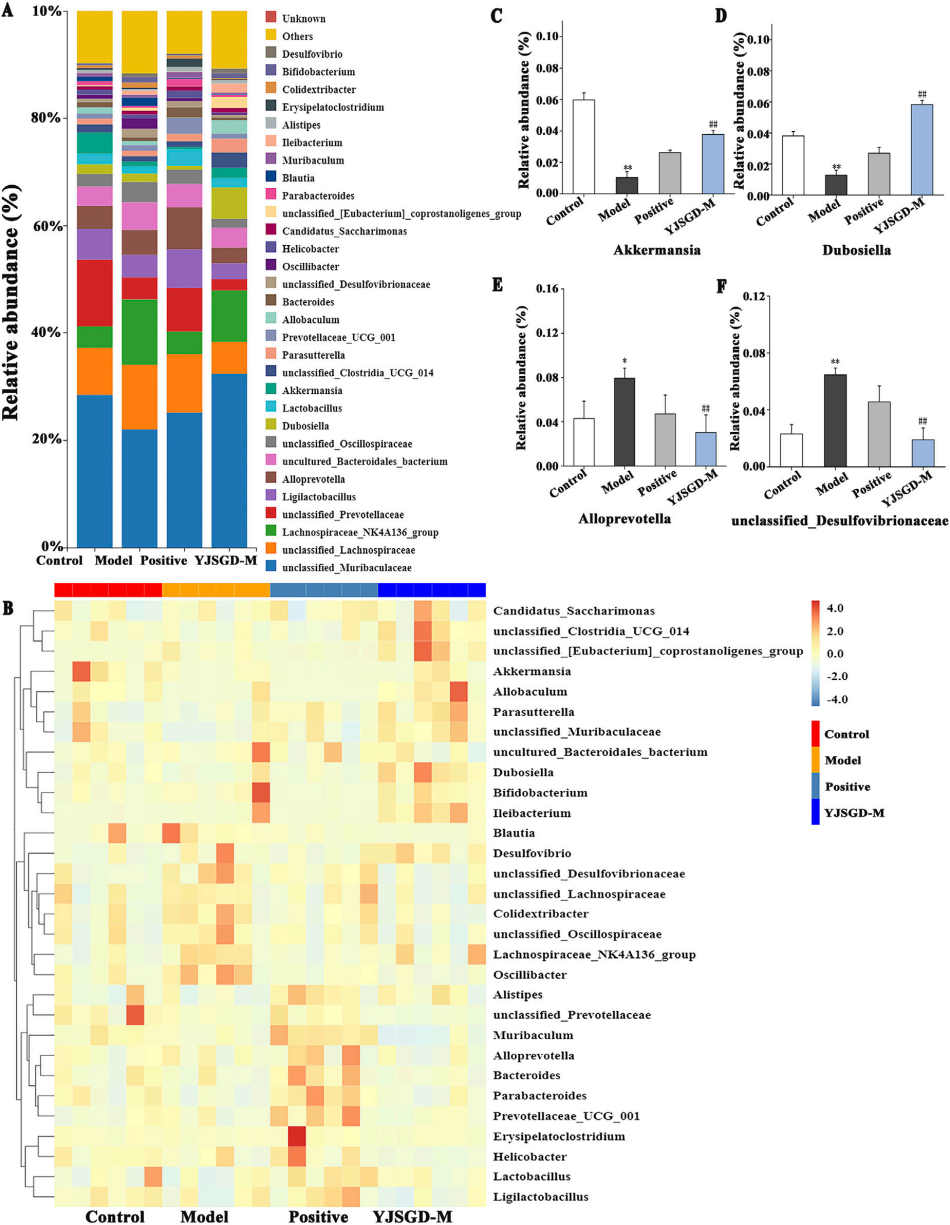
YJSGD alters genus-level microbiota composition. **(A)** Genus-level bar plot; **(B)** Genus-level heatmap; **(C–F)** Signature bacteria. Data are displayed as means ± SD, n = 6. ^*^ *p* < 0.05, ^**^ *p* < 0.01 vs. the control group. ^#^ *p* < 0.05, ^##^ *p* < 0.01 vs. the model group.

In the genus-level analysis, the model group showed a significant reduction in beneficial bacteria compared to the control group, including *Akkermansia*, *Allobaculum, unclassified Muribaculaceae*, *Parasutterella*, *Bifidobacterium*, and *Dubosiella*. Conversely, potentially pathogenic genera such as, Colidextribacter, and unclassified Desulfovibrionaceae were markedly enriched (Figure 6A). Following YJSGD intervention, the abundance of beneficial bacteria including *Akkermansia*, *Dubosiella*, *Bifidobacterium* and *Ileibacterium* increased in abundance, while the levels of potentially pathogenic bacteria decreased. These suggest that YJSGD restores intestinal microecological balance by precisely regulating key bacterial genera (Figures 6B,C).

#### 3.7.3 YJSGD increased SCFAs levels in POF mice

The content of SCFAs in the feces of each group of mice is shown in Table 4. The results indicate that acetic acid, propionic acid, and butyric acid are the primary components of SCFAs in the feces of all groups of mice, while isobutyric acid, valeric acid, isovaleric acid, and hexanoic acid are present in lower concentrations. Compared to the control group, the SCFAs levels in the model group were significantly reduced (*p* < 0.05). Compared with the model group, YJSGD intervention significantly increased fecal SCFAs concentrations in POF mice. Specifically, propionate, butyrate, and isobutyrate levels exhibited marked elevation (*p* < 0.05, *p* < 0.01).

**TABLE 4:** Fecal SCFAs concentrations in mice.

Group	Acetic acid (mmol/L)	Propionic acid (mmol/L)	Butyric acid (mmol/L)	Isobutyric acid (μmol/L)	Valproic acid (μmol/L)	Isovaleric acid (μmol/L)	Hexanoic acid (μmol/L)
Control	0.77 ± 0.11	0.29 ± 0.04	0.19 ± 0.01	15.23 ± 2.20	9.11 ± 1.02	8.89 ± 1.03	20.57 ± 0.68
Model	0.62 ± 0.11	0.16 ± 0.03^*^	0.05 ± 0.01^*^	12.82 ± 1.43	7.09 ± 0.80^*^	7.25 ± 0.75	16.10 ± 2.13
Positive	0.67 ± 0.08	0.18 ± 0.01	0.14 ± 0.01^#^	14.44 ± 1.68	9.26 ± 0.81	9.25 ± 0.35	17.90 ± 2.30
YJSGD-25	0.66 ± 0.09	0.18 ± 0.03	0.17 ± 0.02	22.85 ± 2.47^#^	10.98 ± 1.87^#^	8.66 ± 0.69	23.73 ± 1.96^#^
YJSGD-50	0.69 ± 0.10	0.23 ± 0.04^#^	0.24 ± 0.03^#^	16.90 ± 0.73	13.21 ± 1.71^##^	10.51 ± 1.56^#^	19.59 ± 1.67
YJSGD-100	0.68 ± 0.09	0.19 ± 0.07	0.11 ± 0.02	17.99 ± 1.16	7.99 ± 0.87	8.37 ± 0.76	16.12 ± 2.06

^*^ *P* < 0.05, Model group vs. Control group.

# *P* < 0.05.

##*P* < 0.01 YJSGD, group vs. Model group.

#### 3.7.4 Spearman correlation analysis reveals the association between gut microbiota and SCFAs

**FIGURE 7:**
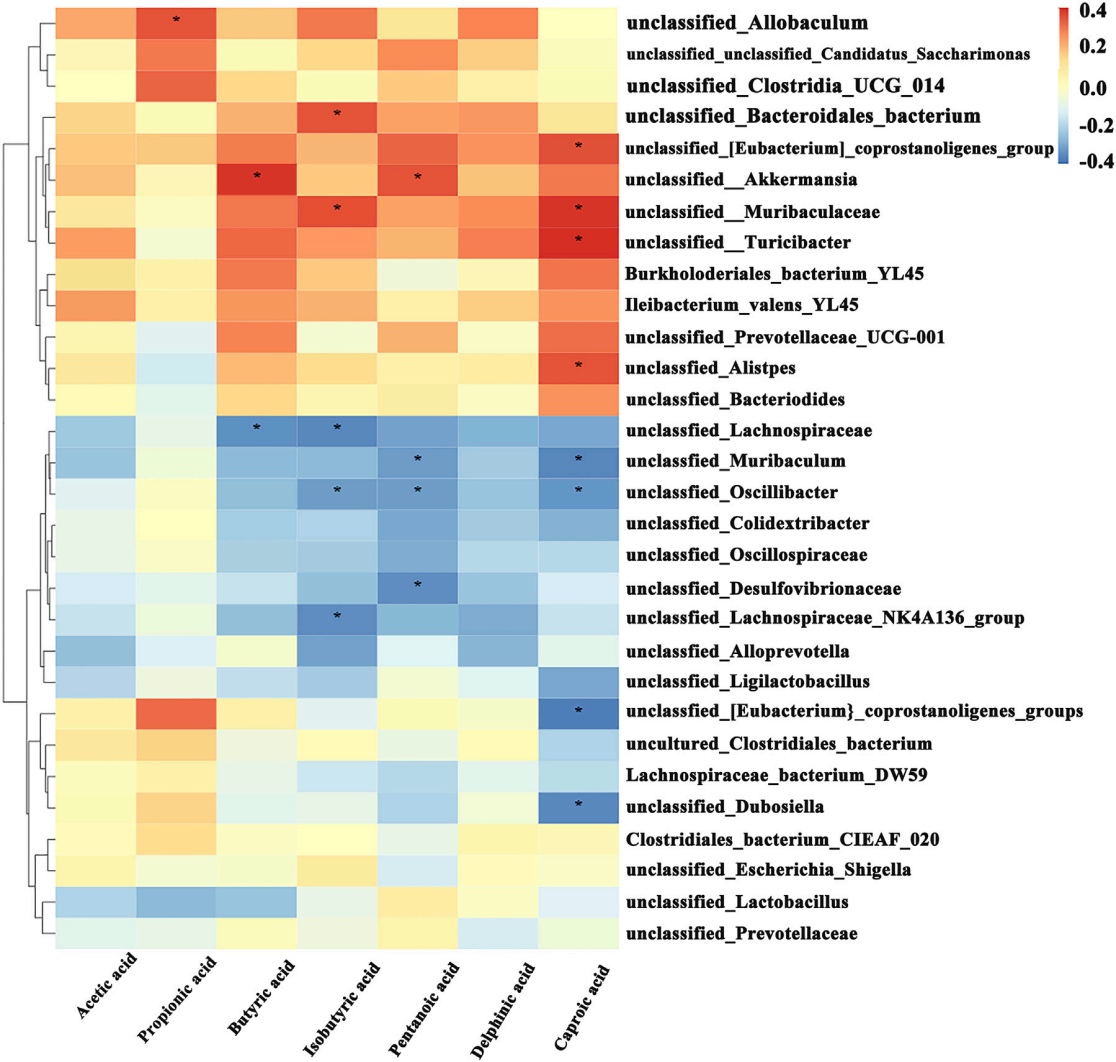
Spearman correlation analysis between SCFAs and gut microbiota (n = 6).

Spearman correlation analysis (Figure 7) demonstrated significant correlations (*p* < 0.05) between specific gut microbial genera and POF-related SCFAs levels. Notably, *unclassified_Allobaculum*, *unclassified_Bacteroidales bacterium*, *unclassified_Akkermansia*, *unclassified_Muribaculaceae*, and *unclassified_Turicibacter* showed significant positive correlations with SCFAs, while *unclassified_*Lachnospiraceae, *unclassified_Muribaculum*, *unclassified_Oscillibacter*, and *unclassified_*Desulfovibrionaceae exhibited significant negative correlations. These findings suggest that YJSGD may ameliorate POF progression by modulating the “gut microbiota-SCFAs” axis.

### 3.8 Effects of YJSGD on metabolites in POF mice

#### 3.8.1 Intergroup metabolite differential analysis

**FIGURE 8:**
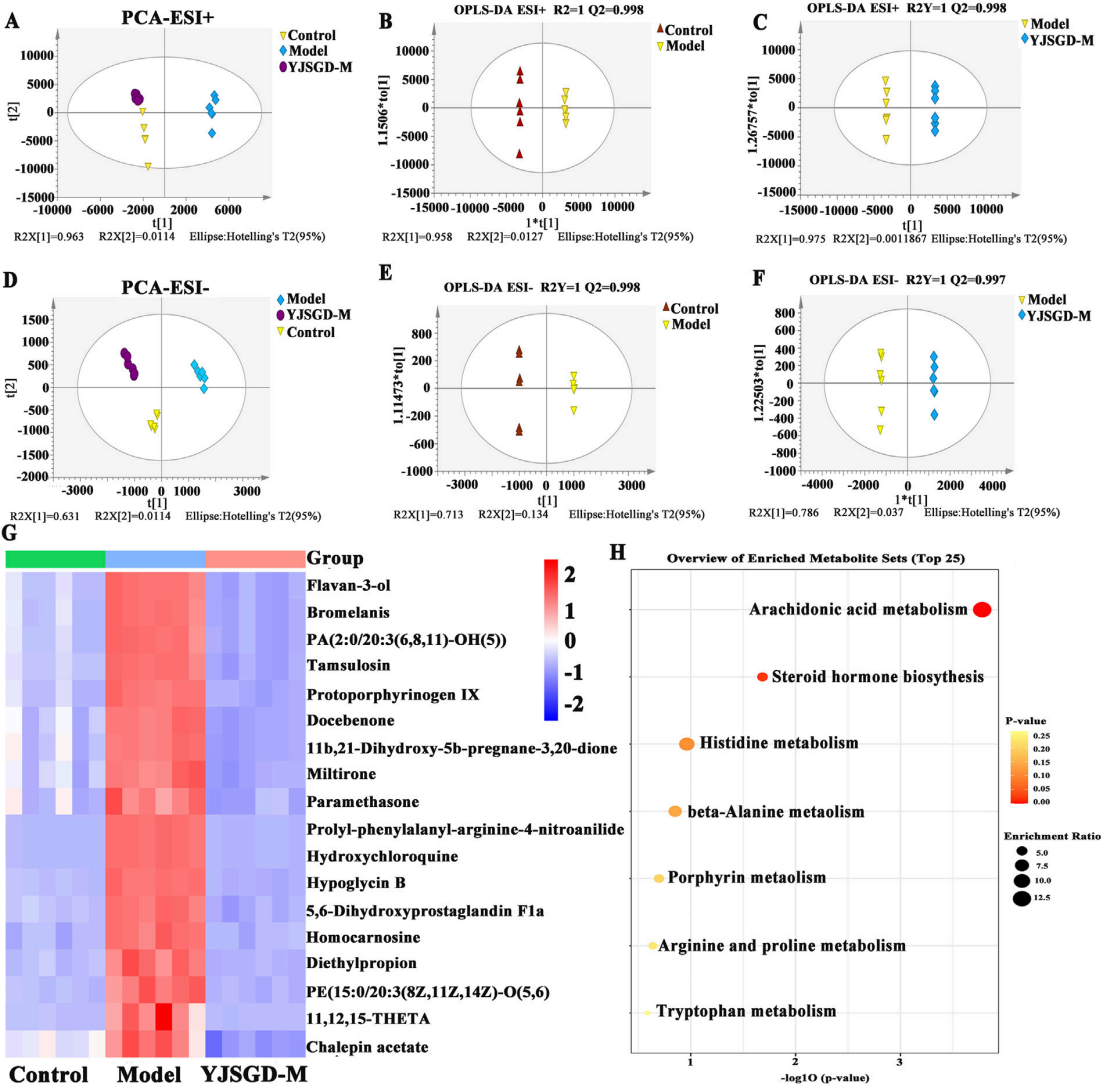
Serum metabolomics analysis (n = 6). **(A)** OPLS-DA analysis of samples in positive ion mode. **(B)** OPLS-DA analysis of control group and model group; **(C)**: OPLS-DA analysis of model group and YJSGD-M group; **(D)**: OPLS-DA analysis of samples in negative ion mode. **(E)** OPLS-DA analysis of control group and model group; **(F)**: OPLS-DA analysis of model group and YJSGD-M group. **(G)**: heat map **(H)**: bubble plot.

Initial preprocessing of the raw MS data from all groups involved noise reduction, baseline correction, normalization, and peak alignment, followed by comprehensive analysis with SIMCA 14.1 software. PCA results (Figures 8A–F) demonstrated that serum samples from the control group formed tight clusters without overlap in the projection space. Although minor intergroup overlap was observed, significant separation distances between differently colored groups indicated distinct metabolic profiles under both positive and negative ion modes. The OPLS-DA analysis of differential blood metabolites in mice revealed distinct separations between the blank control group and model group, as well as between the model group and YJSGD-M group in the score plots, demonstrating significant metabolic differences between each paired comparison.

#### 3.8.2 Differential metabolites screening

This study integrated untargeted metabolomics with bioinformatics analysis to systematically identify differential metabolites and conduct metabolic pathway enrichment, providing a comprehensive characterization of metabolic perturbations in POF mice. Based on the criteria of VIP >1 and *p* < 0.05, a total of 15 common differential metabolites were identified as potential biomarkers under both ESI^+^ and ESI^−^ modes (Figure 8G; Table 5). Notably, YJSGD administration effectively normalized the dysregulated expression levels of these 15 serum metabolites in POF model mice.

**TABLE 5:** Characteristics of metabolic biomarkers and their variations across groups.

NO.	Metabolites	Formula	R.T. (min)	M/Z	Model vs. Control	YJSGD-M vs. Model
1	Tamsulosin	C_20_H_28_N_2_O_5_S	1.349	409.1789	↑	↓
2	Paramethasone	C_22_H_29_FO_5_	1.478	393.205	↑	↓
3	Flavan-3-ol	C_15_H_14_O_2_	1.508	453.2044	↑	↓
4	Bromelains	C_11_H_17_N_2_NaO_3_	1.542	497.23	↑	↓
5	PA (2:0/20:3 (6,8,11)-OH(5))	C_25_H_43_O_9_P	2.762	541.2559	↑	↓
6	Protoporphyrinogen IX	C_34_H_40_N_4_O_4_	6.893	569.3077	↑	↓
7	Hypoglycin B	C_12_H_18_N_2_O_5_	9.592	541.2557	↑	↓
8	Prolyl-phenylalanyl-arginine-4-nitroanilide	C_26_H_34_N_8_O_5_	10.712	537.2618	↑	↓
9	Hydroxychloroquine	C_18_H_26_ClN_3_O	10.815	669.3392	↑	↓
10	PE (15:0/20:3 (8Z,11Z,14Z)-O (5,6))	C_40_H_72_NO_9_P	11.569	740.4832	↑	↓
11	5,6-Dihydroxyprostaglandin F1a	C_20_H_36_O_7_	12.208	389.2531	↑	↓
12	11,12,15-THETA	C_20_H_34_O_5_	12.950	353.2299	↑	↓
13	Diethylpropion	C_13_H_19_NO	14.308	433.2791	↑	↓
14	Homocarnosine	C_10_H_16_N_4_O_3_	17.994	481.2565	↑	↓
15	Chalepin acetate	C_21_H_24_O_5_	22.847	355.1544	↑	↓

Metabolites with “↑/↓” means increased/decreased expression.

#### 3.8.3 Metabolic pathway analysis

To investigate the effects of YJSGD on metabolic pathways associated with blood biomarkers in POF model mice, we performed pathway enrichment analysis of differential metabolites using MetaboAnalyst 5.0 (Figure 8H). KEGG metabolic pathways were screened based on the criteria of VIP >1 and *p* < 0.05, and pathway importance was evaluated through topological analysis: a bubble plot was generated with Pathway Impact as the x-axis and enrichment significance (-log_10_P) as the y-axis. The red bubbles in the upper-right quadrant (with size and color intensity increasing with significance level) indicate key metabolic pathways, where higher -log_10_P values reflect stronger intergroup metabolic associations. Pathway enrichment results demonstrated that the differential metabolites were primarily associated with two core pathways: arachidonic acid metabolism and steroid hormone biosynthesis, with secondary involvement of histidine metabolism and β-alanine metabolism.

#### 3.8.4 Spearman analysis between intestinal flora and differential metabolites

**FIGURE 9:**
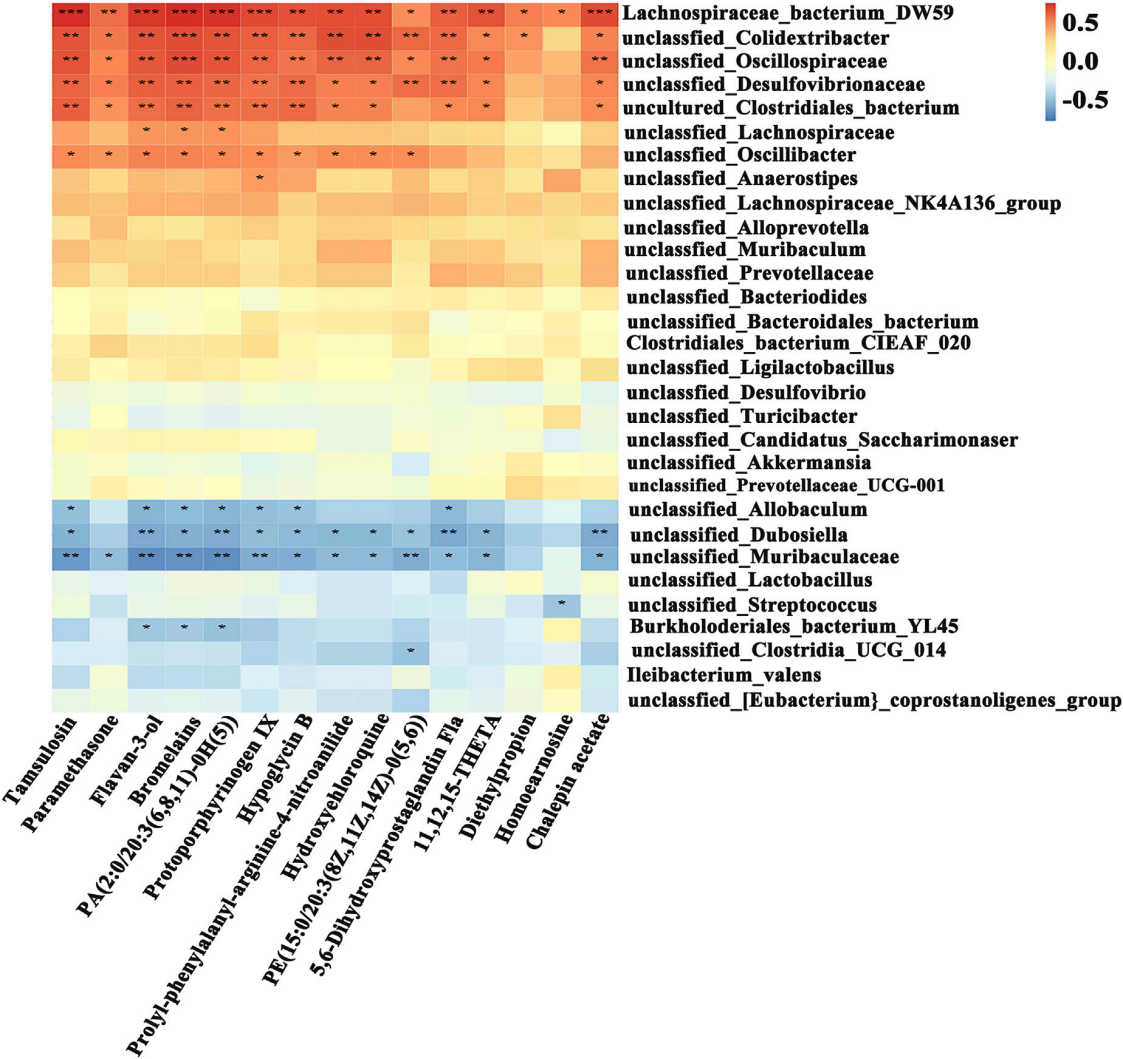
Spearman correlation analysis between gut microbiota and serum biochemical indicators (n = 6).

To elucidate the correlation between gut microbiota and metabolites, this study systematically evaluated the associations of 30 bacterial species with 15 differential metabolites using Spearman correlation analysis (Figure 9). The results revealed significant correlations between specific microbial taxa and metabolites. Notably, Lachnospiraceae*_bacterium_DW59* and *unclassified_Akkermansia* showed positive correlations with Flavan-3-ol, Paramethasone, 5,6-Dihydroxyprostaglandin F1a, and Homocarnosine, whereas *Burkholderiales_bacterium_YL45*, *unclassified_Turicibacter*, and *Ileibacterium_valens* exhibited negative correlations (r < −0.5) with Paramethasone and Prolyl-phenylalanyl-arginine-4-nitroanilide. These findings suggest that YJSGD may ameliorate POF progression by modulating the synergistic interplay between gut microbiota and host metabolites.

## 4 Discussion

POF is a complex clinical syndrome that can lead to a range of health issues, including an increased risk of cardiovascular disease, reduced bone density, significantly decreased fertility, and atrophy of the vulva and vagina (Tsiligiannis et al., 2019). Therefore, exploring safe and effective treatment options for POF has become a key research focus in reproductive medicine both domestically and internationally. In recent years, TCM has demonstrated significant advantages in preventing and treating POF due to its safety and efficacy. This study focused on a compound (YJSGD) composed of 10 traditional botanical drugs. After identifying its major metabolites, the formula was found to significantly restore disrupted menstrual cycle in D-gal-induced POF mice, reverse abnormal serum levels of FSH, LH, and E2, alleviate oxidative stress, and restore gut microbiota homeostasis. These results indicate that YJSGD effectively restores ovarian function and delays the ovarian aging process through multitarget regulation.

Ovarian dysfunction is a prominent feature of POF patients, characterized by hormonal imbalances, reduced follicle numbers, and disrupted menstrual cycles. Studies confirm that TCM can ameliorate these symptoms, comprehensively alleviating POF-related pathological changes and restoring ovarian function. Research by Liang et al. demonstrated that the Wenzhong Bushen formula reversed serum hormonal imbalances, increased the ovarian index, normalized the estrous cycle, restored follicle counts at all developmental stages, and improved ovarian morphology in POF mice, ultimately restoring ovarian function (Liang et al., 2024). Kuntai Capsules combined with hormone therapy can improve ovarian reserve function, increasing the antral follicle count and anti-Mullerian hormone (AMH) levels while reducing FSH levels (Zhang et al., 2024). The Zishen Yutai pills, composed of *Cuscutae Semen*, *Amomum villosum Lour*., and *Polygonum multiflorum Thunb*, improves diminished ovarian reserve function by regulating sex hormone levels and inhibiting ovarian granulosa cell apoptosis (Zhang et al., 2025). The comprehensive, multi-pathway, and multi-target regulatory advantages of the TCM compound for POF may be attributed to its diverse potential active metabolites. Research indicates that epigallocatechin gallate and theaflavins alleviate cyclophosphamide-induced ovarian damage by inhibiting excessive primordial follicle activation and follicular atresia (Chen et al., 2021). Paeoniflorin improves the estrous cycle, serum hormone levels, and the number of antral follicles in mice with reduced ovarian reserve function, restoring E2 synthesis in ovarian granulosa cells (Wu et al., 2023). Hyperoside, found in *C. chinensis*, prevents cyclophosphamide-induced ovarian injury by inhibiting HIF-1α/BNIP3-mediated autophagy, thereby reducing primordial follicle depletion and follicular atresia (Zhu et al., 2022). In this study, our chemical analysis identified major metabolites in YJSGD, a compound comprising ten botanical drugs including *C. officinalis* and *R. glutinosa*, such as hyperoside, morroniside, and paeoniflorin. Compared with the model group, 50 mg/kg YJSGD intervention significantly ameliorated serum estrogen imbalance in POF mice, manifested by a 74.45% increase in E2 levels, alongside 53.17% and 24.03% reduction in FSH and LH levels, respectively.

Oxidative stress damages oocytes, disrupts ovarian tissue structure and function, and accelerate ovarian aging (Gao et al., 2023). Medicinal plants contain various natural antioxidant metabolites. UPLC-Q-TOF-MS analysis identified multiple metabolites in YJSGD, including paeoniflorin, arbutin, hyoscyamine, naringin, mononitroflavone, tetrandrine, asterol, rehmannioside D, luteolin, and ferulic acid. These metabolites exhibit notable efficacy in combating oxidative stress and restoring ovarian function, as supported by established biological evidence. Paeoniflorin promotes murine ovarian development through its capacity to amelirate oxidative stress damage (Wu et al., 2023). Mononitroflavone reduces apoptosis in rat granulosa cells by activating the PI3K/AKT/mTOR pathway, consequently improving cellular oxidative stress resistance (Deng et al., 2021). Furthermore, luteolin potently enhances antioxidant by restoring the functional activity of the Nrf2 pathway (Huang and Zhang, 2021). This study revealed that YJSGD significantly upregulated Sirt1 expression while downregulating Keap1, thereby promoting Nrf2 activation and the subsequent upregulation of downstream HO-1. These coordinated changes suggest that the amelioration of oxidative damage in ovarian tissue and the improvement of ovarian function by YJSGD may involve the activation of the Sirt1/Keap1/Nrf2 signaling axis. Future studies employing Sirt1 inhibitors or genetic approaches are warranted to establish a definitive causal relationship. Furthermore, oxidative stress may trigger inflammation response. Inflammation alters the ovarian microenvironment, leading to ovarian cell dysfunction or death, playing a crucial role in ovarian aging and POI (Huang et al., 2019). Studies indicate that chrysin improves ovarian function by reducing inflammation and oxidative stress in D-gal induced ovarian failure (Li S. et al., 2022); Quercetin significantly mitigates D-gal-induced oxidative stress and inflammation primarily by increasing endogenous antioxidant enzyme activity (such as CAT, SOD, and GSH-Px) and reducing the levels of oxidative productsand pro-inflammatory factors (Chen et al., 2022). In the present work, YJSGD treatment significantly suppressed the secretion of IL-1β, IL-6, and TNF-α, indicating its efficacy in alleviating inflammatory markers expression in POF mice. The findings above have demonstrated the therapeutic effect of YJSGD on POF mice and identified ten major metabolites, several of which have documented multi-faceted activities including ovarian protection, antioxidant, and anti-inflammatory effects, thereby providing direct and valuable candidates for subsequent research.

Mechanistically, YJSGD ameliorates ovarian function by regulating the gut microbiota composition and establishing an efficient SCFAs metabolic network through significant enrichment of key beneficial bacteria such as *unclassified_Akkermansia* (genus *Akkermansia*), *unclassified_Muribaculaceae* (genus *Muribaculaceae*), *Ileibacterium_valens* (genus *Ileibacterium*), and *unclassified_Allobaculum* (genus *Allobaculum*). Among these, *Akkermansia* peoduces acetate and propionate, which not only exert direct anti-inflammatory effects but also serve as substrates for butyrate-producing bacteria, such as *Allobaculum*, *Clostridia* UCG-014, *Ileibacterium*), thereby significantly promoting butyrate production via cross-feeding (Ratajczak et al., 2019).

SCFAs, key bioactive metabolites derived from gut microbiota fermentation, enhance systemic antioxidant defense by directly scavenging free radicals such as superoxide anions and hydroxyl radicals, activating the Nrf2 signaling pathway to upregulate antioxidant enzymes including SOD and GSH-Px, and inhibiting TNF-α/IL-6-induced ROS overproduction through GPR43/41-mediated suppression of NF-κB signaling (Li X. et al., 2022; Wang et al., 2025). Experimentally, YJSGD-M group exhibited increases in fecal acetate, propionate, and butyrate levels by 0.043-fold, 0.447-fold, and 3.17-fold, respectively, compared to the model group. The generated SCFAs, particularly butyrate, activate the Nrf2-Keap1 antioxidant pathway, upregulating the activity of enzymes such as SOD and GSH-Px. At the same time, YJSGD increased the abundance of Actinobacteria, a phylum which contains probiotics such as *Bifidobacterium*. Actinobacteria have been shown to alleviate ovarian inflammation by regulating the Treg/Th17 balance. Concurrently, YJSGD inhibited pro-inflammatory bacteria such as *Blautia* and *Oscillibacter* (Dai et al., 2024).

Non-targeted metabolomics provided further confirmation of its antioxidant and anti-inflammatory effects. This study found that in the model group mice, the levels of endogenous differential metabolites such as 5,6-dihydroxyprostaglandin F1a, 11,12,15-THETA, and various oxidized phospholipids (PA (2:0/20:3 (6,8,11)-OH(5)), and PE (15:0/20:3 (8Z,11Z, 14Z)-O (5,6))) were significantly elevated. These substances serve as clear markers of overactivated arachidonic acid metabolism and also function as pro-inflammatory lipid peroxidation products (Imig, 2020). The increased levels of these metabolites in the model group, combined with the dysregulation of homocarnosine, collectively reflect a state of chronic inflammation and oxidative damage in the ovaries. The accumulation of protoporphyrinogen IX in POF mice indicates mitochondrial dysfunction and impaired heme synthesis, which not only leads to a cellular energy crisis but also directly inhibits steroid hormone biosynthesis by affecting cytochrome P450 enzyme activity (Xia et al., 2024). These interconnected metabolic abnormalities disrupt the follicular development environment and compromise follicular survival capacity, ultimately contributing to premature ovarian failure. Following YJSDG intervention, the levels of these aberrant metabolites normalized. Importantly, positive correlations between *Allobaculum/unclassified Muribaculaceae*/*/Dubosiella* and the above endogenous differential metabolites imply these microbial communities may regulate host metabolism through metabolic transformation or synergistic effects (Ursell et al., 2014). Specifically, the model group exhibited disruptions in both arachidonic acid metabolism and steroid hormone biosynthesis pathways. This was accompanied by elevated levels of pro-inflammatory metabolites, including prostaglandin derivatives. Conversely, *Burkholderiales_bacterium* may disrupt steroid homeostasis via hydroxylation/glucuronidation modifications, potentially accelerating intestinal degradation of corticosteroids like paramethasone (Szaleniec et al., 2018). Their pro-inflammatory activity could exacerbate intestinal barrier damage, triggering systemic oxidative stress and accelerating ovarian functional decline. Collectively, YJSGD restores ovarian function not only through direct repair but also by remodeling gut microbiota, establishing SCFAs metabolic networks, activating the Nrf2-Keap1 antioxidant pathway, and suppressing pro-inflammatory factors to modulate host metabolism.

## 5 Conclusion

This study comprehensively demonstrates that YJSGD exerts multi-dimensional therapeutic effects against D-gal-induced premature ovarian failure through integrated modulation of ovarian function, oxidative homeostasis, inflammatory responses, and the gut-ovary axis. YJSGD effectively restored ovarian cyclicity and follicular development while rebalancing reproductive hormone levels. The treatment also alleviated oxidative damage, which was correlated with the activation of the Sirt1/Nrf2 pathway, as evidenced by the subsequent enhancement of antioxidant enzymes (SOD, CAT, GSH-Px) and reduction of lipid peroxidation. The decoction simultaneously attenuated systemic inflammation via downregulation of pro-inflammatory cytokines (IL-1β, TNF-α, and IL-6) and reestablished gut microbiota equilibrium with increased beneficial SCFAs production. Notably, serum metabolomics revealed YJSGD’s profound regulation of amino acid, lipid, and energy metabolism pathways. The identification of characteristic major metabolites, including Rehmannioside D, coupled with these multi-systemic effects, provides mechanistic validation for YJSGD’s traditional application in ovarian dysfunction while highlighting its unique capacity to concurrently address reproductive and metabolic dimensions of POF pathogenesis. These findings position YJSGD as a novel multi-target therapeutic agent bridging botanical drugs and modern systems biology approaches for ovarian protection. However, there are some limitations in this study. First, although the correlation between YJSGD intervention and gut microbiota remodeling has been established, it would be valuable to employ germ-free animal models and fecal microbiota transplantation experiments to further verify the necessity of gut microbiota in mediating the therapeutic effects of YJSGD. Second, exogenous supplementation experiments could be conducted to assess whether specific metabolites are able to replicate the therapeutic efficacy of YJSGD, thereby clarifying their functional roles within the “gut microbiota-metabolite-ovary” axis. Finally, applying network pharmacology and molecular docking to construct a “metabolite-target-pathway” network, followed by experimental validation, will help elucidate the multi-target synergistic mechanisms of YJSGD.

## Data Availability

The data presented in the study are deposited in the NCBI (SRA) repository, accession number PRJNA1393598.
