# Supplementary material for: Yangjing Shugan decoction targets the Sirt1/Nrf2 antioxidant pathway and gut microbiota-metabolite axis to protect against premature ovarian failure
Source: Front Pharmacol. 2026 Jan 16;16:1722692. doi: 10.3389/fphar.2025.1722692 (PMC12855410; doi:10.3389/fphar.2025.1722692)
Supplement: Supplementary file 1 [file Supplementaryfile1.docx]

**Supplementary material**

**
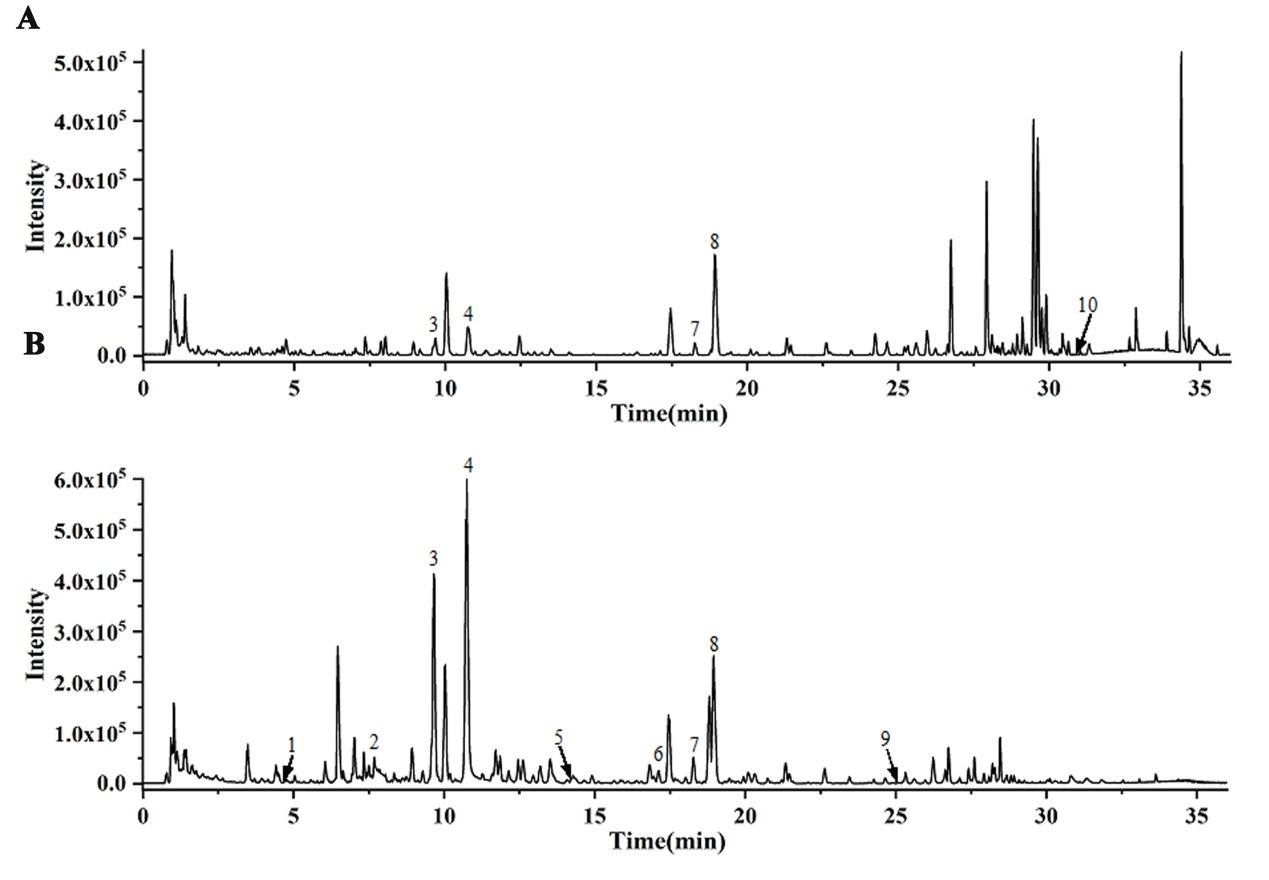
**

**Fig. S1 Total ion chromatography of YJYGD by LC-MS/MS analysis: (A) negative ion modes; (B) positive ion modes. 1:** **Rehmannioside D; 2:** **Morroniside; 3:** **Loganin; 4:** **Paeoniflorin; 5:** **Ferulic acid; 6:** **Hyperoside; 7:** **Specnuezhenide; 8:** **Hesperidin; 9:** **Luteolin; 10:Ursolic acid.**


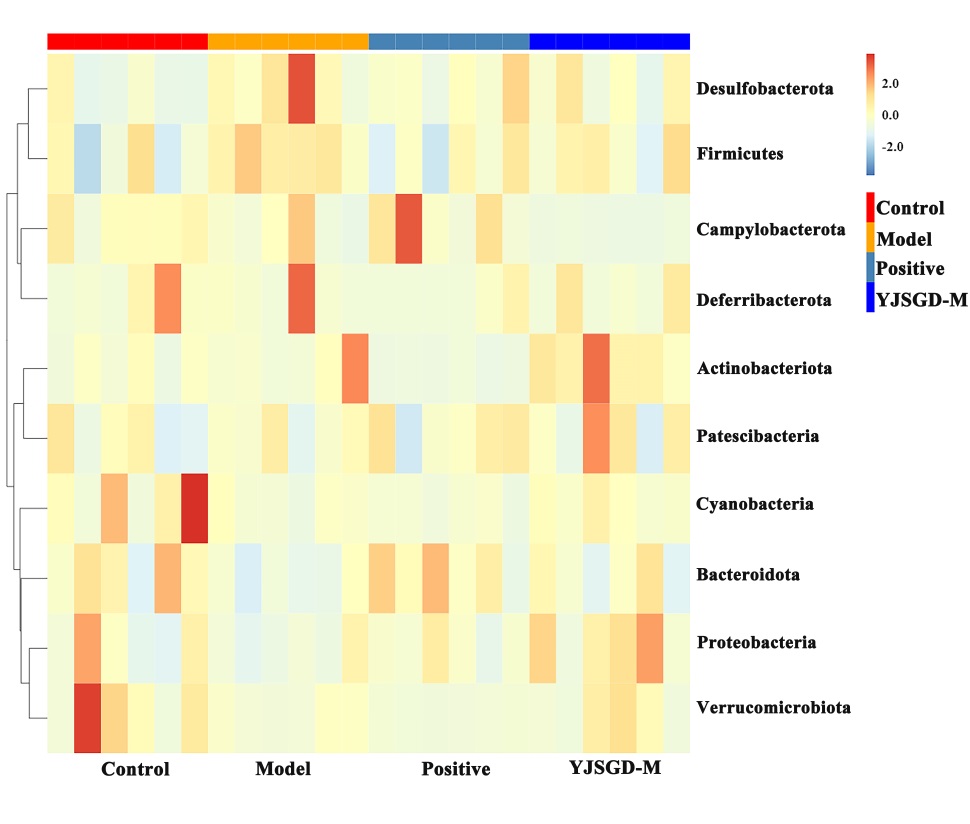


**Fig. S2** Fecal microbiota heatmap
